# Selective nociceptor activation in volunteers by infrared diode laser

**DOI:** 10.1186/1744-8069-7-18

**Published:** 2011-03-22

**Authors:** Alexander Z Tzabazis, Michael Klukinov, Sonia Crottaz-Herbette, Mikhail I Nemenov, Martin S Angst, David C Yeomans

**Affiliations:** 1 Department of Anesthesia, Friedrich Alexander University, Erlangen, Germany; 2 Department of Anesthesia, Stanford University, Stanford, CA, USA; 3 Lasmed, LLC, Mountain View, CA, USA

## Abstract

**Background:**

Two main classes of peripheral sensory neurons contribute to thermal pain sensitivity: the unmyelinated C fibers and thinly myelinated Aδ fibers. These two fiber types may differentially underlie different clinical pain states and distinctions in the efficacy of analgesic treatments. Methods of differentially testing C and Aδ thermal pain are widely used in animal experimentation, but these methods are not optimal for human volunteer and patient use. Thus, this project aimed to provide psychophysical and electrophysiological evidence that whether different protocols of infrared diode laser stimulation, which allows for direct activation of nociceptive terminals deep in the skin, could differentially activate Aδ or C fiber thermonociceptors in volunteers.

**Results:**

Short (60 ms), high intensity laser pulses (SP) evoked monomodal "pricking" pain which was not enhanced by topical capsaicin, whereas longer, lower power pulses (LP) evoked monomodal "burning" pain which was enhanced by topical capsaicin. SP also produced cortical evoked EEG potentials consistent with Aδ mediation, the amplitude of which was directly correlated with pain intensity but was not affected by topical capsaicin. LP also produced a distinct evoked potential pattern the amplitude of which was also correlated with pain intensity, which was enhanced by topical capsaicin, and the latency of which could be used to estimate the conduction velocity of the mediating nociceptive fibers.

**Conclusions:**

Psychophysical and electrophysiological data were consistent with the ability of short high intensity infrared laser pulses to selectively produce Aδ mediated pain and of longer pulses to selectively produce C fiber mediated thermal pain. Thus, the use of these or similar protocols may be useful in developing and testing novel therapeutics based on the differential molecular mechanisms underlying activation of the two fiber types (e.g., TRPV1, TRPV2, etc). In addition, these protocol may be useful in determining the fiber mediation of different clinical pain types which may, in turn be useful in treatment choice.

## Background

Numerous studies have shown that there are two main classes of pain sensing neurons in the skin and other peripheral tissues: myelinated Aδ and unmyelinated C nociceptor[1,2]. There are many distinctions between these two types of nociceptive afferents [3,4]. Activation of these afferents evokes distinct sensations: Aδ fiber mediated pain is typically described as rapid, pricking or sharp, and localized whereas C fiber mediated pain is usually described as a diffuse, burning or aching sensations that outlast stimulus duration[5-7].

Although separate activation of Aδ and C fiber nociceptors, demonstrated electrophysiologically, has been achieved through the use of radiant skin heating in rats[4], these methods are not optimal for testing in humans. In the last two decades human studies have often used lasers as tools for evaluation of nociception and the integrity of nociceptive pathways in experimental pain models and neurological diseases[8-20,32].

Coherent radiation, as from a laser, is not a stimulus found in nature. However, as with radiant heat, lasers provide the advantage over contact stimulators of providing a purely thermal, as opposed to mixed, stimulation. In addition, lasers allow brief pulses (μs to ms) with very fast rise time. Beyond these advantages, diode lasers provide uniform skin heating from approximately 50 to 600 μm deep and a high level of pulse repeatability. Because the surface does not need to be overheated to provide conductive heating of deeper nociceptors (as with CO2 and Thulium lasers), diode lasers provide a degree of added safety over these devices. Diode laser stimuli have been used for *in vivo *cutaneous stimulation of free nerve endings in humans and rodents and for *in vitro *activation of TRPV1 and TRPV2 channels in cultured dorsal root ganglia neurons and HEK273 cells[18,21-23]. These experiments have indicated that brief, high heating rate diode laser pulses can selectively activate myelinated Aδ fiber nociceptors in rats and produce pricking pain in humans, whereas low heating rate, longer pulses can preferentially activate unmyelinated C fibers in rats and produce burning pain in humans. However, it is not known whether these different pulse parameters will differentially activate Aδ or C fibers in humans. To investigate this question, we used a combination of psychophysical and electrophysiological (cortical evoked potentials) to investigate whether brief pulses would produce both singular pricking pain and a cortical activation pattern characteristic of Aδ fiber activation, as well as whether long pulses would produce singular burning pain and cortical activation consistent with C fiber activation. In addition, to provide complimentary evidence, we investigated the differential effects of topical capsaicin, which selectively sensitizes TRPV1 expressing, mostly unmyelinated nociceptors[4].

## Results

### Repeatability and intensity response

**Figure 1:**
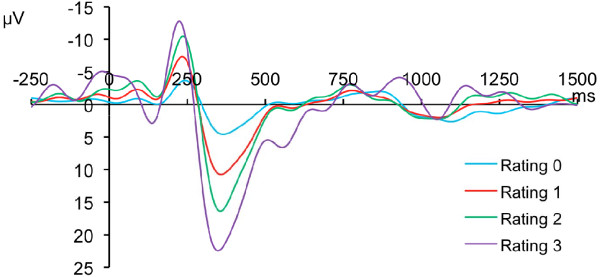
**Cortical Evoked Potential Evoked by 60 ms High Intensity Diode Infrared Laser Pulses**. Each trace represents grand average of EEG response of 10 subjects subjected to 15 pulses of each of 4 intensities (3 painful intensities, 1 sub pain threshold). Traces show distinct earlier negative (up) phase and positive (down) phases the amplitude of which are directly correlated to the numeric pain rating evoked for that stimulus.

Intensities of SP stimuli below pain threshold produced a "touch" sensation. Above pain threshold, SP stimulation with a numerical rating (NRS) rating of 1 to 3, pain was described as "pricking" and "singular/monomodal", suggestive of Aδ nociceptor mediation. Higher intensities produced NRS ratings greater than 3, with which the incidence of bimodal pricking-burning sensations increased. SP above pain threshold produced a sinusoidal LEP with a first negative peak appearing between 225 and 246 ms after the laser pulse was applied to the finger, and a positive peak between 363 and 378 ms (Figure 1). Subtracting the 60 ms pulse duration, these values are consistent with a conduction velocity (CV) between 4.1 to 4.5 m/s - in the Aδ range. SP below pain threshold ("touch") produced LEP at similar latencies but with much less sharp peaks and lower amplitudes. For each subject, both positive (r^2^= 0.98) and negative (r^2^= 0.98) peak amplitudes were significantly (p < 0.05) correlated with the NRS rating. Stimuli were highly repeatable, as neither pain ratings nor LEP peak amplitudes were significantly different between the average of the first 5 stimuli and the last (p < 0.05 in each case).

**Figure 2:**
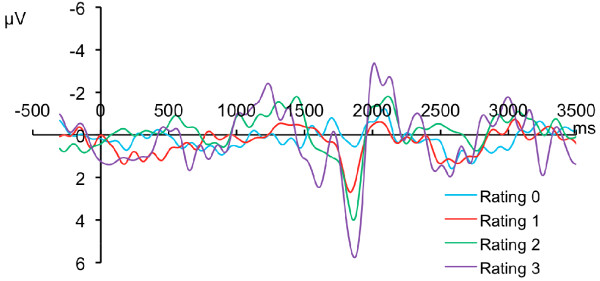
**Cortical Evoked Potential Evoked by 1.5 s Low Intensity Diode Infrared Laser Pulses**. Each trace represents grand average of EEG response of 10 subjects subjected to 15 pulses of each of 4 intensities (3 painful intensities, 1 sub pain threshold). Traces are aligned with the start of the pulses and show small earlier (first) negative (up) phase and positive (down) phases followed by another (second) negative phase. The amplitude of the positive and second negative waves were directly correlated to the numeric pain rating evoked for that stimulus.

LP stimuli at or above pain threshold (NRS 1-3) produced monomodal/singular "burning" pain suggestive of C fiber mediation. Intensities below pain threshold were perceived as "warm". When stimulus intensities that evoked moderate pain (NRS 4-6) were applied, bimodal pricking-burning pain was sometimes reported. LP above pain threshold produced a sinusoidal LEP with a small (first) negative peak at about 1220 to 1460 ms following the 1.5 s pulse followed by a positive peak in between 1858 to 1887 ms and a larger (second) negative peak between 2036 to 2112 ms (Figure 2). Using this stimulus, the skin temperature rises at about 20°C/sec from a controlled baseline of 33°C, creating a delay of about 600 ms for skin heating to threshold temperatures. The remaining component of the latency is due to the conduction time from the periphery as well as time of central processing necessary to produce a cortical potential. Thus, the latency to the end of the threshold level stimulus, after subtracting the temperature rise time, yields a CV of 1.041 - 1.614 m/s, consistent with C fiber mediation. There was a significant (p < 0.05) correlation with the NRS rating and the positive (r^2 ^= 0.66) as well as the second negative (r^2 ^= 0.89) peak. As with SP, LP stimuli were highly repeatable, as neither pain ratings nor LEP peak amplitudes were significantly different between the average of the first 5 stimuli and the last (p < 0.05 in each case).

### Capsaicin Sensitization

**Figure 3:**
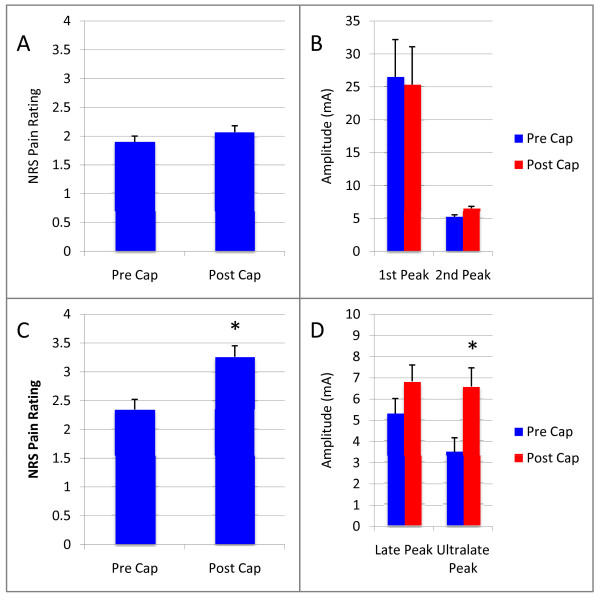
**Effect of Topical Capsaicin on Pain Ratings and Amplitude of Cortical Potentials Evoked by Infrared Diode Laser Pulses**. A. 1% capsaicin did not affect numerical pain ratings evoked by a 60 ms suprathreshold laser stimulus. B. Similarly capsaicin did not affect evoked potential responses to short laser pulses. C. Capsaicin did however, induce a significant (p < 0.05) hyperalgesia for pain evoked by longer (1.5 s) laser pulses. D. Similarly, topical capsaicin increased the amplitude of cortical evoked responses to 1.5 s laser pulses, significantly (p < 0.05) in the case of the second negative peak.

**Figure 4:**
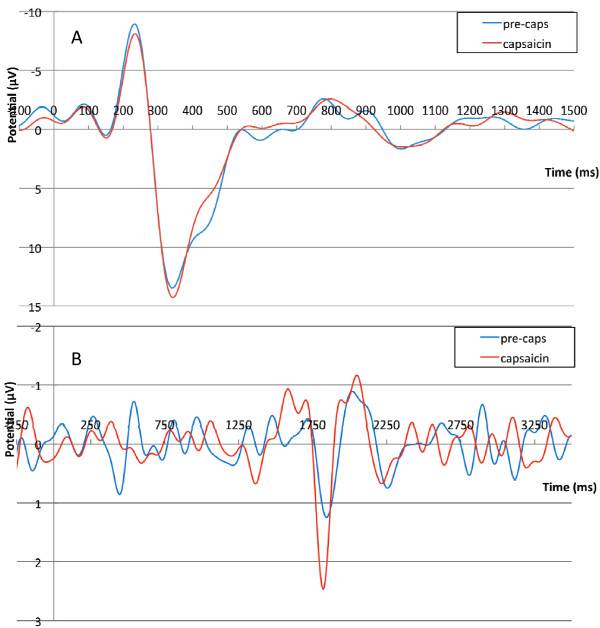
**Effect of Topical Capsaicin on Laser Evoked Potentials**. A. Topical pretreatment with 1% capsaicin did not affect grand average (across 7 subjects) LEP evoked by a suprathreshold short (60 ms) laser pulses. B. In contrast, topical capsaicin clearly enhanced cortical responses to the suprathreshold long (1.5 s) laser pulses.

The effects of topical capsaicin was tested in separate sessions in seven of the ten subjects. Topical treatment with capsaicin significantly (p < 0.05) decreased the threshold current necessary to induce pain sensation (NRS 1) for LP, but did not change the threshold for SP (p > 0.05) and increased the pain NRS evoked by a given suprathreshold intensity of stimulation (p < 0.05) (Figure 3a). Similarly, for a given intensity of LP, both the first and second negative peaks were increased by topical capsaicin, the second effect significantly (p < 0.05) (Figure 3b). There was no effect of capsaicin on either pain rating (Figure 3c), or LEP amplitude (Figure 3d) for responses to SP. Traces in Figure 4 are grand average LEPs across subjects demonstrating the lack of effect of capsaicin on SP response (Figure 4a) and the facilitating effect of capsaicin on LEP evoked by LP (Figure 4b).

### Conduction Velocity

Recording LEP after elbow stimulation was substantially problematic, such that only two subjects had sufficiently clean recordings to work with. For these the average latency shift for LP stimulation of the elbow vs the hand (30 cm) was 218 ms for the negative peak, which is consistent with C fiber CV of 1.38 m/s. The peak shift for SP was too short to reliably assess using this method.

## Discussion

This study sought to investigate whether brief, intense, high heating rate pulses emitted by a diode infrared laser would evoke pain mediated selectively or preferentially and repeatedly by the activation of myelinated (Aδ) thermonociceptors, whereas lower rates of skin heating produced by lower intensity laser pulses would produce pain mediated selectively or preferentially by the activation of unmyelinated (C) thermonociceptors. We assessed psychophysical responsiveness and cortical potentials evoked by two laser skin heating parameters in human volunteers. For SP laser stimuli close to pain threshold, pain was described as monomodal and sharp or pricking, whereas LP laser stimuli were described as monomodal and burning.

This finding of monomodal pain modalities appears to be unique in the literature, as laser stimuli usually evoke double pain sensations indicative of activation of both A and C thermonociceptors [24-26]. Monomodal sensation has been achieved with other lasers [10] and with high-rate skin contact skin heating[26], but it is not clear that the sensation perceived was pain, but rather may be monomodal warm mediated by the activation of unmyelinated warm fibers[26]. The ability to evoke monomodal pain with an infrared diode laser is likely due to the homogenous heating of epidermal and dermal tissue (for both hairy and glabrous skin), up to 600 microns from the surface[18]. The range of depths of (at least unmyelinated) nociceptive terminals in the skin is 40-570 μm[27], thus fairly well matching the range of homogenous direct tissue heating of infrared diode lasers. Other lasers (e.g., CO_2_) and high rate contact heating predominantly heat the surface, wherefrom surface heat is conducted to underlying tissue - a process that requires time and overheating of the surface[24]. Simultaneous direct heating of epidermal as well as deeper nerve terminals allows for differential activation based on the activation properties of those terminals - as opposed to the conductive properties of the tissue.

Near threshold pain evoked by LP appears to be mediated by the selective activation of C fiber nociceptors; pain evoked by SP appears to be mediated by the activation of Aδ nociceptors. LP-evoked pain is monomodal "burning" and, along with cortical evoked potentials, is enhanced by topical capsaicin, and demonstrates a latency shift representative of a CV of less than 1.7 m/s - all characteristics of C fiber thermonociceptors[3-5,28,29]. SP-evoked pain is characterized as monomodal "pricking", is not enhanced by capsaicin, and has an estimated CV of around 4.5 m/s - all characteristics of Aδ thermonociceptors[4,7,28].

LEP produced by these two stimulus types were also distinct. LEP evoked by suprathreshold SP were sinusoidal, insensitive to topical capsaicin, and produced an estimated conduction velocity in the Aδ range. LEP evoked by suprathreshold LP were also sinusoidal, but were enhanced by topical capsaicin and gave a conduction velocity estimate in the C fiber range. Thus, LEP measurements agreed with psychophysics in demonstrating a fiber-selective differential activation by short vs. long pulses of infrared laser light. It is possible that warm fibers also contributed to the LEP recorded in response to LP as the threshold for warm-sensitive C fibers is reached and surpassed as the temperature rises with this stimulus ~38-40°C[30]. In addition, the conduction velocity of C fiber warm receptors is typically in the range of 2.5 m/s, well above those measured in the current study[31]. Interestingly, Magerl used a CO_2 _laser to separate Aδ vs C fiber mediated cortical responses[32] in volunteers. However, the stimulus used to activate C fibers (40°C) was below that typically activating C nociceptors, and the conduction velocity estimated in these studies (2.4-2.8 m/s) was close to that measured for human C warm receptors[31] suggesting that the cortical response recorded in this study may have been mediated or dominated by warm fibers, rather than thermonociceptors.

## Conclusions

The results of these experiments indicate that short, high intensity laser pulses can be used to selectively produce Aδ mediated pain and LEP in humans and that longer duration, lower intensity pulses can be used to selectively produce C mediated pain and LEP in humans. These protocols may be useful then, in evaluating differential pharmacologic effects and physiologic mechanisms of these two distinct pain types.

## Methods

### Subjects

After approval by the Stanford Institutional Review Board and after informed consent, 10 healthy volunteers, 5 female and 5 male, aged 21-50 (median 31) years participated in this study. All subjects gave informed consent to participate in the study, which was approved by the Stanford Institutional Review Board.

### Laser stimulation

Two different infrared diode laser (Lass 10, Lasmed LLC, Mountain View, CA) settings were used: 1) a short pulse (SP): 60 ms, 0.3 mm^2^, heating ramp up to 600 C°/s, or 2) a long pulse (LP): 1.5 s, 40 mm^2^, heating ramp up to 20 C°/s were applied to 10-20 spots on the hairy skin of the dorsum of the 2^nd ^through 5^th ^fingers (i.e., not the thumb) of human volunteers. SP was hypothesized to preferentially activate myelinated thermonociceptors, whereas LP was hypothesized to mainly stimulate unmyelinated thermonociceptors, respectively. Baseline skin temperature was measured periodically in between stimulation and maintained at 33 ± 0.5°C using a heating pad placed on the surface of the skin between stimulus sessions and under the hand during stimulus sessions.

### Laser Evoked Potentials (LEP)

#### EEG collection

Laser evoked potentials (LEP) were recorded using Ag-AgCl surface scalp electrodes and Acquire 4.3 software with a 36-electrode Quik-Cap (both NeuroScan Inc, El Paso, Texas). Data was acquired simultaneously from 32 EEG channels (in accordance with Enhanced 10-20 International system), and 2 EOG channels - vertical VEOU and horizontal VEOL, wth AFz as a ground electrode, and A2 as a common reference, using NuAmps 40 Channel Digital DC EEG amplifier (NeuroScan Inc, El Paso, Texas). The impedance was generally maintained below 5 kΩ. The signals were bandpass (analog 0.1-70 Hz) and notch (60 Hz) filtered in real time, and digitized at a rate of 1000 Hz.

#### EEG Processing

EEG data then were imported and pre-processed in EEGLAB, a free open-source toolbox running under MatLab environment. Continuous data was bandpass-filtered from 0.5 to 20 Hz, and epochs containing LEP (1000 ms prior to stimulus and 4000 ms post-stimulus) were subsequently extracted and baseline corrected to 1 s before stimulus onset. High-amplitude noise contaminated channels and epochs were rejected upon visual inspection. In order to remove ocular artifacts while preserving useful signal, we used independent component analysis (ICA) to decompose EEG into a number of statistically independent components, and then subtract noise from the original data [5]. For better results two runs of ICA were performed: first epochs containing non-stereotype artifacts were identified and rejected [2], then ocular artifacts were removed from the data. Cz channel data were extracted from each data set, and indexed with a custom database. LEP waveforms were computed by averaging epochs pulled from the database per request.

### Repeatability

To test for repeatability of laser stimuli, threshold stimulus intensities (measured in supplied current) for evoking pain were measured for both pulse types (in a randomly order). Then, LEPs were recorded while each subject received 20 stimuli of either SP or LP, set at 15% above the threshold stimulus intensity, and separated by an approximately 30 s interstimulus interval. After each successive pulse, subjects were asked to give a forced-choice between descriptors (sharp or burning; monomodal or multimodal) as well as a pain rating on a 0-10 scale (0 = no pain, 1 = threshold level pain, 10 = intolerable pain).

### Intensity response

Pain threshold was determined using a random staircase method of assessment of pain thresholds. The laser current necessary to evoke a pain rating of 1 on an 11 point NRS scale was measured at 10-20 sites on the dorsal skin surface of the 2nd, 3rd, 4th, and 5th fingers (i.e., not the thumb) of each volunteer. The average of laser currents necessary to produce a rating of "1" was used to establish the pain threshold. To determine an intensity-effect relationship, both randomly selected SP and LP were then applied with increasing stimulus intensities to different areas of the dorsum of on hand with at least 30 s between stimuli while LEPs were recorded. Subjects were asked to rate the pain immediately after each stimulus. Stimulus intensities were increased in 100 mA (~ 300 mW) increments and each stimulus intensity was presented 4 times within a session. Intensity increases were continued until the subject reached a level of strong pain (rating > 3). Intensity response relationships were then determined for both evoked pain and LEP.

### Capsaicin Sensitization

To provide evidence of nociceptor selectivity for pain evoked by LP or SP, changes in LEP and pain sensitivity were tested after topically applying 30 μl of capsaicin (1% in H_2_O/Ethanol, 50/50 v/v), to the finger of 7 subjects after establishing baseline pain thresholds. 20 minutes after applying capsaicin, thresholds were re-established and subjects were asked to rate the level of pain evoked by the pretreatment threshold current. Significant sensitization was determined by using a one-tailed t test to compare pain thresholds and LEP amplitudes before and after treatments, as well as pain levels evoked by a given stimulus level (pretreatment threshold).

Non-parametric statistics were used to determine whether there was a significant change in descriptors between those given after the first 5 stimuli and those given after the last 5. Analyses of variance were used to detect a significant shift in the average pain rating and LEP amplitude following the first 5 and the last 5 stimuli.

### Conduction Velocity

In order to determine conduction velocity and thus provide additional evidence for selective activations of Aδ versus C fibers, in some subjects, after applying 15 SP or LP stimuli, set at a power that was 15% above threshold, the same stimuli were applied to the elbow, while LEP were recorded. The latency from stimulus onset to LEP peak were measured and used to calculate an estimated conduction velocity of the sensory fibers underlying responses to these stimuli.

## Competing interests

MIN is the CEO of LasMed, LLC, manufacturer of the laser used in this study. None of the other authors declare competing interests.

## Authors' contributions

AT contributed to the data analysis and manuscript preparation; MK contributed to the EEG data processing and data analysis; SC-H collected much of the data and aided in initial analysis; MIN provided expertise in laser stimulation and to the analysis of data; MSA contributed to the design and analysis of the study; DCY provided overall guidance to the project, contributed to data analysis and manuscript preparation. All authors read and approved the manuscript.
